# Nonasthmatic Eosinophilic Bronchitis: An Overlooked Cause of Chronic Cough in Children

**DOI:** 10.7759/cureus.107552

**Published:** 2026-04-22

**Authors:** Badar Al Dhouyani, Ahmed Abushahin, Mutasim Abu-Hasan

**Affiliations:** 1 Pediatric Pulmonology, Sidra Medicine, Doha, QAT

**Keywords:** bronchoalveolar lavage, children, chronic cough, eosinophilia, non-asthmatic eosinophilic bronchitis

## Abstract

Non-asthmatic eosinophilic bronchitis (NAEB) is an inflammatory airway disease that causes chronic cough, responds well to inhaled steroids, and can be misdiagnosed as asthma because of the pathophysiologic overlap. Unlike asthma, NAEB is not associated with airway hyperresponsiveness. NAEB has not been previously described in children. We report a case of a child with chronic cough, eosinophilic airway inflammation, and a negative methacholine challenge test, strongly suggestive of a diagnosis of NAEB.

The patient was a 10-year-old boy who was hospitalized with a 2-year history of chronic productive cough and exertional dyspnea, which did not respond to repeated treatment with oral antibiotics or inhaled bronchodilators. On examination, he appeared tachypneic with low O2 saturation (89%) on room air. Chest auscultation revealed bilateral crackles. WBC, ESR, and CRP were elevated. Peripheral eosinophil count and Immunoglobulin E (IgE) level were normal. Pulmonary function testing (PFT) demonstrated a combined obstructive and restrictive pattern with air trapping. Fractional exhaled nitric oxide (FeNO) was normal. Chest CT showed airway wall thickening and bilateral small centrilobular nodules, with no evidence of bronchiectasis or interstitial lung infiltrates. Flexible bronchoscopy revealed diffuse thick airway secretions. Bronchoalveolar lavage (BAL) fluid analysis showed a high eosinophil count of 40%. BAL cultures were negative. The patient was initially treated with IV antibiotics and airway clearance. He was then started on inhaled steroids using budesonide/salmeterol MDI (160/25 mcg) because of the airway eosinophilia. Within a few days of treatment with inhaled steroids, the patient’s symptoms improved significantly. He was weaned off O2. The inflammatory markers normalized. Chest CT showed resolution of the abnormal infiltrates. Repeat PFT 4 months later was normal. Methacholine challenge testing was performed twice after discontinuing inhaled steroids, and both tests showed no airway hyperresponsiveness.

NAEB is a possible cause of chronic cough in children that can mimic asthma. Bronchoscopy and a methacholine challenge test may be needed for diagnosis.

## Introduction

Non-asthmatic eosinophilic bronchitis (NAEB) is a chronic airway disease characterized by eosinophilic inflammation and presents with chronic productive cough that responds well to corticosteroids [1]. Although NAEB shares some clinical and pathophysiological features with type 2 asthma, it is distinguished from asthma by the absence of airway hyperresponsiveness [2].

NAEB was first described by Gibson PG et al. in 1989 in nonsmoking adults presenting with chronic productive cough and corticosteroid-responsive sputum eosinophilia [3]. With increased testing of airway inflammation via induced sputum, NAEB has gained recognition as a distinct cause of chronic cough in adults [2,4]. Airway eosinophilia, defined as eosinophils constituting more than 2.5% of cells in induced sputum or bronchoalveolar lavage (BAL) fluid, is a hallmark of NAEB. Crucially, the absence of airway hyperresponsiveness, as demonstrated by a negative methacholine challenge, differentiates NAEB from asthma [4,5].

Patients with NAEB typically present with chronic productive cough lasting longer than 2 months. Chest X-ray is usually normal. Pulmonary function tests (PFTs) may be normal or may show airway obstruction, which is not reversible with bronchodilators [4,5]. The exact etiology of NAEB remains unclear. Environmental and occupational exposures have been implicated in some cases. Allergic responses to air pollutants may trigger NAEB [5]. However, identifying specific environmental triggers or specific inhaled allergens is not required for NAEB diagnosis [6].

In children, NAEB has not been recognized as a distinct disease entity or as a cause of chronic cough. The underuse of induced sputum analysis and bronchoscopy in the routine evaluation of pediatric patients with chronic cough contributes to this under-recognition. Furthermore, children with chronic cough are often empirically treated with inhaled corticosteroids (ICS), which are an effective treatment for both NAEB and asthma. As a result, many pediatric NAEB cases may be misdiagnosed as asthma and treated successfully with inhaled steroids, while the true diagnosis of NAEB remains undetected. However, it is very important to distinguish between NAEB and asthma because of the differences in prognosis and management strategies of the two entities.

We report a case of a child who presented with chronic intractable cough, exertional dyspnea, irreversible small airway obstruction, and airway eosinophilia detected by flexible bronchoscopy and BAL. The diagnosis of NAEB was confirmed by ruling out other causes of chronic cough, airway obstruction, and airway eosinophilia. The diagnosis of NAEB was also supported by the complete resolution of clinical, radiological, and PFT changes with inhaled steroids. More importantly, asthma was ruled out by negative methacholine challenge testing.

## Case presentation

The patient is a 10-year-old boy who was admitted to a tertiary pediatric hospital with a two-year history of progressively worsening chronic cough productive of yellow sputum. He also complained of exertional dyspnea, with no history of wheezing. His symptoms persisted despite multiple courses of oral antibiotics and daily inhaled bronchodilators. His past medical history was unremarkable. Family history of asthma, eczema, or allergic rhinitis was negative. The patient had one sibling who was diagnosed with eosinophilic esophagitis. There was no history of exposure to pets, tobacco smoke, or other environmental irritants.

On examination, the patient appeared tachypneic, with a respiratory rate of 32 breaths per minute, but there were no signs of respiratory distress such as retractions, nasal flaring, or use of accessory muscles. Oxygen saturation was 89% on room air, requiring 5 L/min of supplemental oxygen via face mask to maintain saturation above 95%. Chest auscultation revealed diffuse bilateral crackles, with normal and equal air entry and no wheezing. The remainder of the physical examination was unremarkable.

**Figure 1:**
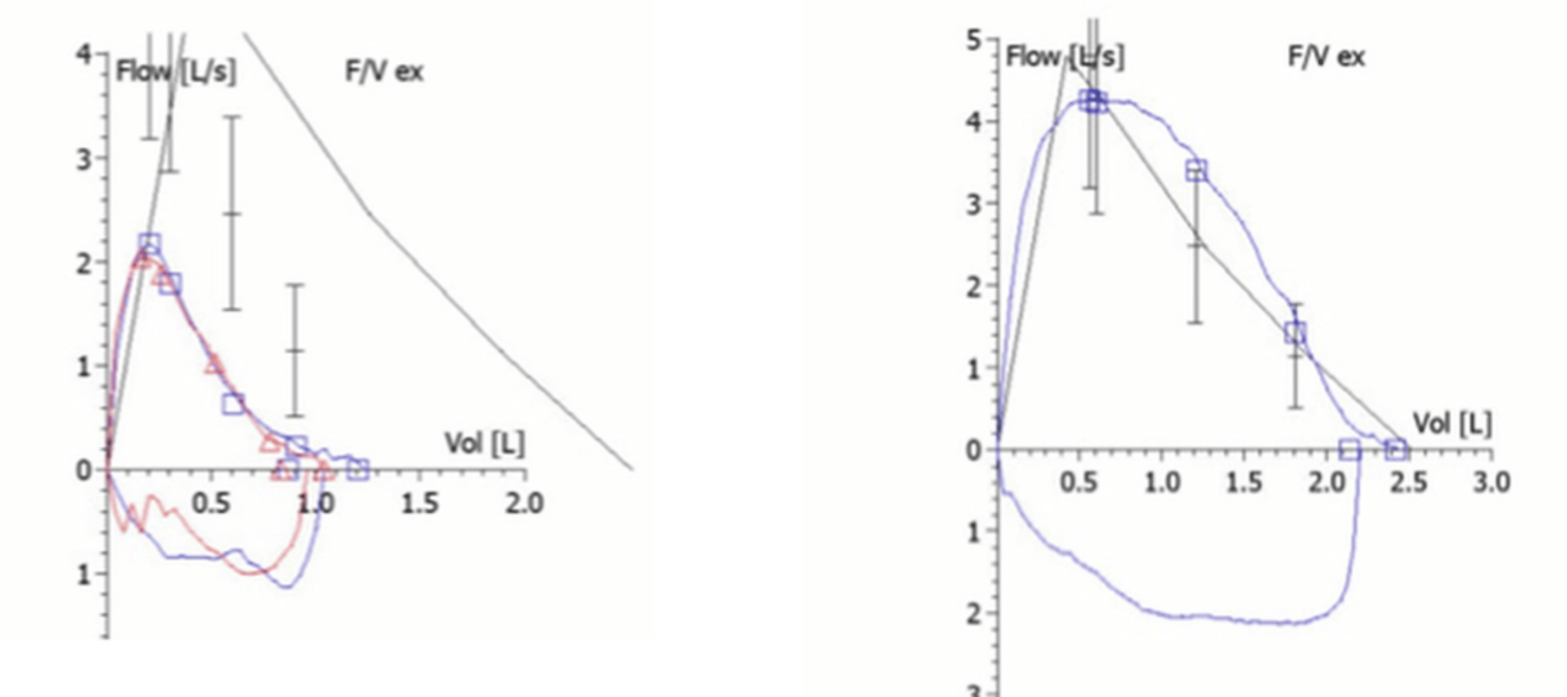
(1-A) Flow-volume loop before treatment showing reduced vital capacity and a scooping pattern (blue), with no reversibility after bronchodilator administration (red). (1-B) Flow-volume loop after treatment showing significant improvement in vital capacity, with marked reduction in the scooping pattern.

Pulmonary function testing showed a combined restrictive and obstructive pattern with no reversibility after bronchodilator administration (Figure 1). Lung volume measurements showed air trapping, with a high residual volume (RV) of 205% predicted and a high RV/total lung capacity (TLC) ratio of 57% (Table 1). TLC was normal. Lung diffusion capacity (DLCO) was normal at 121% of predicted, corrected for hemoglobin and alveolar volume. Fractional exhaled nitric oxide (FeNO) was also within the normal range at 7.2 parts per billion (ppb).

**Table 1:** Pulmonary function test results before and after ICS/LABA treatment, showing marked improvement in FVC, FEV1, and VC, with marked decreases in RV and RV/TLC. FVC: Forced vital capacity; FEV1: Forced expiratory volume in 1 second; FEF25-75%: Forced expiratory flow between 25% and 75% of vital capacity; sRaw: Specific airway resistance; VC: Vital capacity; TLC: Total lung capacity; RV: Residual volume; ICS/LABA: Inhaled corticosteroid/long-acting beta2-agonist.

Measured variable	Before treatment	4 months after treatment
FVC, L (% predicted)	1.20 (48)	2.41 (96)
FEV1, L (% predicted)	0.86 (40)	2.13 (99)
FEV1/FVC (%)	72	103
FEF25-75%, L/s (% predicted)	0.57 (23)	3.01 (122)
sRaw, kPa·s	1.48 (277)	0.68 (127)
VC, L (% predicted)	1.24 (47)	2.4 (91)
TLC, L (% predicted)	2.9 (84.4)	3.0 (85.7)
RV, L (% predicted)	1.7 (205)	0.6 (71)
RV/TLC (%)	57.9	19.7

Blood testing showed an elevated ESR of 105 mm/hour and a CRP level of 111.6 mg/L. WBC count was elevated at 16.6 × 10⁹/L, but the absolute eosinophil count was normal (0.1 × 10⁹/L). Antinuclear antibodies, including p-ANCA and c-ANCA, were negative. Total IgE and specific IgE titers to common aeroallergens were within normal limits. Serum immunoglobulins (IgA, IgM, IgG, and subclasses) and lymphocyte subsets were normal. QuantiFERON test was negative. Sweat chloride was <10 mmol/L.

**Figure 2:**
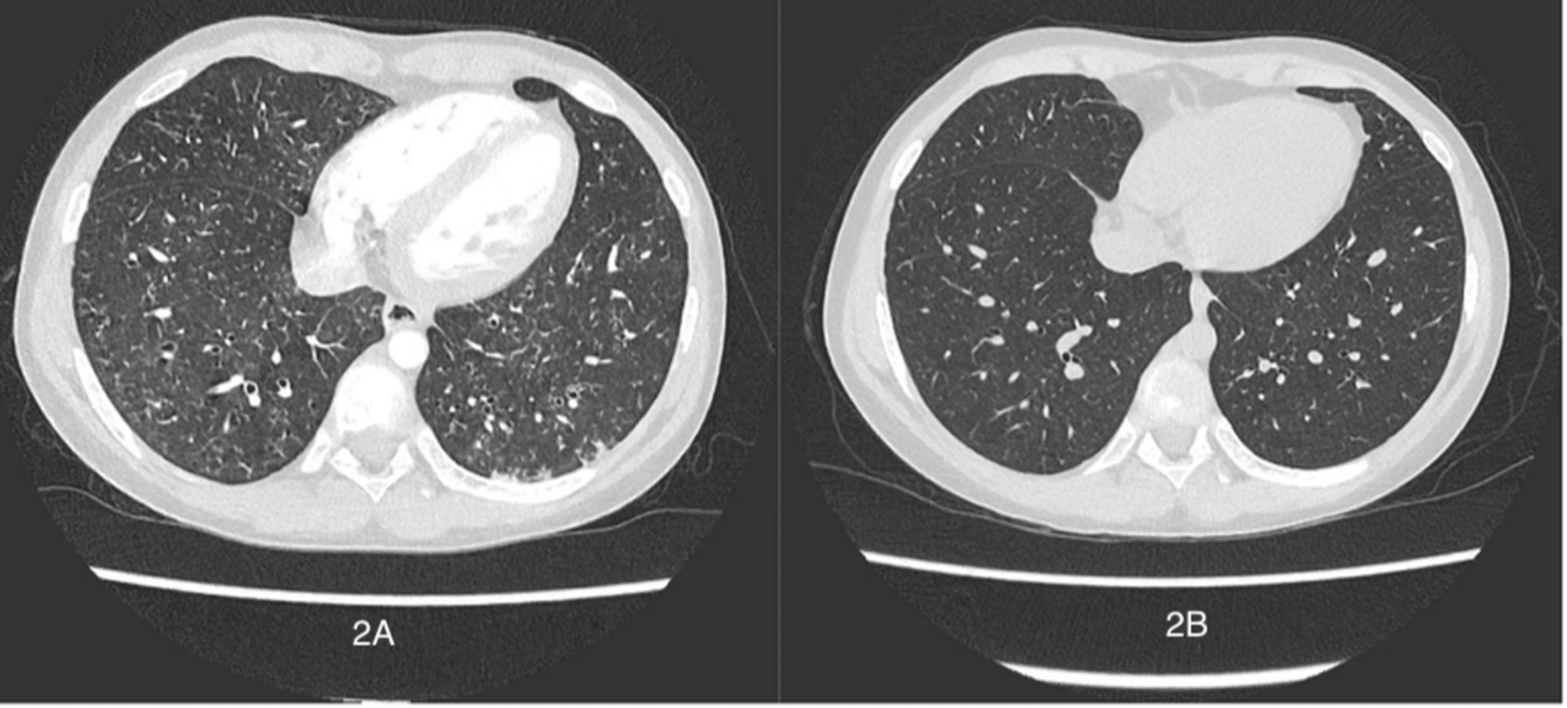
(2-A) Chest CT before treatment with inhaled corticosteroids, showing diffuse centrilobular nodular infiltrates and bilateral peribronchial thickening, with no evidence of bronchiectasis or interstitial ground-glass infiltrates. (2-B) Chest CT after treatment, showing complete resolution of the abnormal infiltrates.

Chest X-ray revealed mild hyperinflation and increased airway markings with diffuse bilateral nodular infiltrates, but no consolidation or atelectasis. High-resolution chest CT showed airway wall thickening, bilateral small centrilobular nodules, and tree-in-bud opacities (Figure 2A). There was no evidence of bronchiectasis or ground-glass infiltrates. CT scan findings were consistent with inflammatory airway disease rather than interstitial lung disease.

Flexible bronchoscopy revealed diffuse, copious, thick airway secretions. BAL fluid analysis showed marked eosinophilia (40% of total BAL cell count). BAL cultures were negative for bacteria and fungi.

The patient was initially treated with intravenous amoxicillin/clavulanate, nebulized salbutamol, and 3% hypertonic saline. Subsequently, an ICS/long-acting beta-agonist (LABA) combination (budesonide 125 mcg/salmeterol 25 mcg), at two puffs twice daily via metered-dose inhaler, was added based on the bronchoscopic findings of airway eosinophilia. After starting inhaled steroids, the patient showed dramatic clinical improvement within a few days, with significant reduction in cough, sputum production, and dyspnea, along with complete resolution of crackles on auscultation. He was successfully weaned off supplemental oxygen, and the inflammatory markers normalized.

The patient was discharged home with a working diagnosis of NAEB versus asthma. He was continued on ICS/LABA therapy for two months, followed by a gradual taper over the next two months. He remained symptom-free, as reported at his 4- and 6-month follow-up visits. Repeat spirometry demonstrated significant improvement in forced vital capacity (FVC), forced expiratory volume in 1 second (FEV₁), and lung volumes (Figure 1B, Table 1). Follow-up CT scan revealed complete resolution of the previous infiltrates (Figure 2B). In order to confirm the diagnosis of NAEB and rule out asthma, the patient underwent two methacholine challenge tests one and three weeks after stopping inhaled steroids. Both tests showed no airway hyperresponsiveness, with PC20 >16 mg/mL in each test. Therefore, the diagnosis of NAEB was confirmed.

## Discussion

We report a case of a 10-year-old boy with NAEB who presented with a two-year history of persistent productive cough and exertional dyspnea. Extensive evaluation ruled out other causes of chronic cough. A markedly elevated eosinophil count in BAL fluid raised suspicion for NAEB. The diagnosis of asthma was excluded based on two negative methacholine challenge tests showing no airway hyperresponsiveness.

NAEB is widely recognized in adults as a common cause of chronic cough, accounting for 10-30% of specialist referrals [2,7]. However, NAEB remains widely under-recognized as a disease entity in children [2]. Chronic productive cough is the main presenting symptom of NAEB [2,5]. Eosinophilic inflammation, which is the main pathologic feature of the disease, causes increased cough reflex sensitivity in patients with NAEB [5,8]. Airway eosinophilia is not easily detected in children, since induced sputum and bronchoscopy are not routinely performed. This may explain why NAEB in children is under-recognized. NAEB can be easily misdiagnosed in children as other disease entities that cause chronic cough with or without airway eosinophilia, including atopic asthma, eosinophilic pneumonitis, allergic bronchopulmonary aspergillosis, tracheobronchomalacia, protracted bacterial bronchitis, and bronchiectasis [9].

NAEB and atopic asthma (type 2 asthma) share common clinical and immunological similarities, including chronic cough, airway eosinophilia, and elevated levels of IL-4, IL-5, and other inflammatory mediators [5,8]. However, there are key pathological distinctions. Asthma is characterized by mast cell infiltration of airway smooth muscle (ASM), while NAEB involves mucosal and submucosal mast cells without ASM involvement [1]. This difference may explain the lack of airway hyperresponsiveness in NAEB as opposed to asthma. Superficial mast cell infiltration in NAEB can contribute to heightened cough reflex sensitivity via release of tussive mediators [1,8].

Diagnosing NAEB requires exclusion of other causes of chronic cough. Essential diagnostic tools include clinical evaluation, radiologic imaging, spirometry, and assessment of airway inflammation. Our patient underwent extensive workup to exclude alternative diagnoses other than asthma, including bronchiectasis, allergic bronchopulmonary aspergillosis (ABPA), protracted bacterial bronchitis, and eosinophilic pneumonitis. ABPA was considered unlikely in our case given the negative history of asthma in the past, the absence of bronchiectasis on chest CT, the normal total IgE levels, and the absence of Aspergillus sensitization. Infectious etiologies of chronic cough such as protracted bacterial bronchitis were also ruled out by the negative BAL cultures and the past history of negative response to antibiotics. Idiopathic eosinophilic pneumonitis or eosinophilic pneumonitis due to parasitic infection or drug-induced reaction was considered very unlikely due to the absence of a history of exposure, normal eosinophil count in peripheral blood, and the absence of interstitial lung infiltrates on chest CT scan. More importantly, eosinophilic pneumonitis and parasitic lung infections require specific treatment with systemic steroids and antiparasitic medications and do not improve on inhaled steroid alone, as our patient did.

No typical radiological abnormalities have been described in NAEB. In most cases, a normal chest X-ray and/or chest CT scan is expected. If abnormal, increased bronchial markings or bronchial wall thickening may be present, indicating airway pathology [9]. Our patient had centrilobular nodules with tree-in-bud opacities in addition to bronchial wall thickening. These findings are non-specific but indicate the presence of airway pathology and airway inflammation, which can be explained by NAEB in our case. This imaging pattern, however, can also be associated with other disease entities that cause infectious and noninfectious airway inflammation. These other disease entities were ruled out in our patient, as explained above. In addition, our patient had complete radiological resolution following anti-inflammatory therapy, which further supports a reversible eosinophilic airway inflammation such as asthma or NAEB as the cause of the findings. CT scan showed no evidence of bronchiectasis or ground-glass opacities suggestive of ABPA or related to interstitial lung disease.

PFT in our patient showed a combined obstructive and restrictive pattern. Airway inflammation causing mucus impaction with centrilobular lung infiltrates can cause the obstructive pattern found in our patient. The presence of air trapping, as demonstrated by the high RV and high RV/TLC but with normal TLC, can explain the restrictive pattern (i.e., decreased FVC) found in our patient. Soon after the patient was started on inhaled steroids, which is the treatment for NAEB, the lung function completely normalized. This response to inhaled steroids strongly suggests that the PFT findings were all due to NAEB.

Based on published guidelines for chronic cough diagnosis and treatment, routine use of flexible bronchoscopy is not recommended [10]. In our case, flexible bronchoscopy was performed due to the atypical clinical, PFT, and radiological findings requiring exclusion of alternative diagnoses other than asthma or respiratory tract infection. Flexible bronchoscopy and BAL detected airway eosinophilia, which raised suspicion for the diagnosis of NAEB. Airway eosinophilia can be detected via induced sputum in the adult population as an alternative to BAL, with eosinophils >2.5% considered abnormal [5]. However, our patient could not produce sputum, so we proceeded with flexible bronchoscopy.

FeNO can be helpful in patients with NAEB to exclude type 2 asthma, since high levels are typical in asthma. However, FeNO has low diagnostic value for NAEB [10]. In our case, FeNO was normal (7.2 ppb), while BAL eosinophils were markedly elevated (40%), which was more consistent with NAEB than asthma. Acute eosinophilic pneumonia is another differential diagnosis for eosinophilic airway inflammation. Unlike NAEB, it presents acutely with systemic symptoms, diffuse alveolar infiltrates, and elevated peripheral eosinophils [11,12]. Our patient lacked these features and responded well to inhaled steroids, unlike eosinophilic pneumonia, which typically requires systemic steroids. A comparison of clinical, radiological, physiological, and biological features of NAEB, asthma, and eosinophilic pneumonia is summarized in Table 2. As no single feature is diagnostic, NAEB remains a diagnosis of exclusion.

**Table 2:** Clinical, radiological, physiological, and biological characteristics of atopic asthma (high T2 asthma), non-asthmatic eosinophilic bronchitis (NAEB), and acute eosinophilic pneumonia. NAEB: Non-asthmatic eosinophilic bronchitis; T2: Type 2; PFT: Pulmonary function test; FeNO: Fractional exhaled nitric oxide.

Feature	Atopic Asthma (High T2 asthma)	Non-Asthmatic Eosinophilic Bronchitis (NAEB)	Acute Eosinophilic Pneumonitis
Clinical presentation			
Cough	Common	Common	Common
Wheezing	Likely	Unlikely	Unlikely
Shortness of breath	Likely	Unlikely	Likely
Chest imaging			
Bronchial/peribronchial infiltrates	Likely	Likely	Unlikely
Parenchymal infiltrates	Unlikely	Unlikely	Likely
PFT findings	Obstructive pattern, reversible with bronchodilators	Obstructive pattern, not reversible with bronchodilators	Restrictive respiratory pattern
Airway hyperresponsiveness (methacholine challenge test)	Present	Absent	Absent
FeNO	High	Low	High
Eosinophilia	Likely	Unlikely	Likely
Response to treatment with inhaled steroids	Likely	Likely	Unlikely

Negative methacholine testing is essential in ruling out asthma as an alternative cause of airway eosinophilia and in confirming the diagnosis of NAEB. In our case, the absence of airway hyperresponsiveness was confirmed twice by negative methacholine challenge testing. Both tests were performed one week and three weeks after complete discontinuation of inhaled steroids to rule out the possibility that the patient had asthma and that the airway hyperresponsiveness was suppressed by the ICS.

The family history of eosinophilic esophagitis in our patient’s sibling raises the possibility of a broader eosinophilic disease spectrum, suggesting that comorbid eosinophilic disorders should be considered in pediatric NAEB evaluations.

ICS or ICS/LABA combinations are the mainstay of treatment for NAEB. Most patients improve symptomatically and demonstrate reduced sputum eosinophils with treatment [2,13]. Montelukast may be added to ICS therapy [1,8]. The optimal treatment duration is unclear; however, Zhan et al. recommend continuing ICS for at least two months after symptom resolution to reduce relapse risk [13].

Our patient was previously treated with bronchodilators and multiple courses of oral antibiotics with no improvement. During admission, he showed dramatic improvement with ICS in addition to antibiotic and bronchodilator therapy, which strongly suggests that the patient’s response was specific to ICS. He was discharged home on a two-month course of ICS, followed by tapering, with sustained clinical and radiologic resolution.

Biologic therapies, including anti-IL-4 and anti-IL-5 agents, are effective in other eosinophilic conditions such as asthma and eosinophilic pneumonitis [14], but their role in NAEB remains unstudied. Until data emerge, their use is not recommended outside severe or refractory cases. The long-term natural history of pediatric NAEB is not known. In adults, outcomes vary: some recover completely, while others develop persistent symptoms or even progress to asthma [15]. In a 10-year study, 9% of NAEB patients developed asthma, 66% had persistent symptoms, and only one patient achieved sustained remission [16]. Another large study suggested that most patients respond well to ICS [17]. The effect of ICS on airway eosinophilia is not well defined. In one study, only 21% of patients had normalized sputum eosinophilia after one year of treatment [18]. Patients with persistent airway obstruction were less likely to improve. Long-term follow-up is essential to monitor for asthma progression or airway remodeling [8]. This case highlights the need for more pediatric-specific NAEB studies to define clinical features, outcomes, and optimal therapies. Future trials should also investigate the utility of biologics in severe or refractory pediatric cases.

## Conclusions

NAEB can occur in children, as has been widely described in adults. However, NAEB in children can be easily unrecognized or misdiagnosed. Our case underscores the importance of recognizing NAEB as a potential cause of chronic cough in children. Key diagnostic features include elevated eosinophils in BAL or sputum and the absence of airway hyperresponsiveness. Because of the clinical overlap between NAEB, asthma, and other eosinophilic lung conditions, a thorough evaluation is essential to prevent misdiagnosis, ensure appropriate treatment, and improve patient outcomes.
