# Correction: Nonasthmatic Eosinophilic Bronchitis: An Overlooked Cause of Chronic Cough in Children

**DOI:** 10.7759/cureus.c430

**Published:** 2026-05-08

**Authors:** Badar Al Dhouyani, Ahmed Abushahin, Mutasim Abu-Hasan

**Affiliations:** 1 Pediatric Pulmonology, Sidra Medicine, Doha, QAT

This article has been corrected to add Ahmed Abushahin and Mutasim Abu-Hasan as authors as they were erroneously omitted from the original version.

